# A phylomedicine approach to understanding the evolution of auditory sensory perception and disease in mammals

**DOI:** 10.1111/eva.12047

**Published:** 2013-03-28

**Authors:** John D Kirwan, Michaël Bekaert, Jennifer M Commins, Kalina T J Davies, Stephen J Rossiter, Emma C Teeling

**Affiliations:** 1 UCD School of Biology and Environmental Science & UCD Conway Institute of Biomolecular and Biomedical Research, University College DublinDublin, Ireland; 2 Institute of Aquaculture, University of StirlingScotland, UK; 3 School of Biological and Chemical Sciences, Queen Mary University of LondonLondon, UK

**Keywords:** hearing, phylomedicine, positive selection, purifying selection

## Abstract

Hereditary deafness affects 0.1% of individuals globally and is considered as one of the most debilitating diseases of man. Despite recent advances, the molecular basis of normal auditory function is not fully understood and little is known about the contribution of single-nucleotide variations to the disease. Using cross-species comparisons of 11 ‘deafness’ genes (*Myo15*, *Ush1 g*, *Strc*, *Tecta*, *Tectb*, *Otog*, *Col11a2*, *Gjb2*, *Cldn14*, *Kcnq4*, *Pou3f4*) across 69 evolutionary and ecologically divergent mammals, we elucidated whether there was evidence for: (i) adaptive evolution acting on these genes across mammals with similar hearing capabilities; and, (ii) regions of long-term evolutionary conservation within which we predict disease-associated mutations should occur. We find evidence of adaptive evolution acting on the eutherian mammals in *Myo15*, *Otog* and *Tecta*. Examination of selection pressures in *Tecta* and *Pou3f4* across a taxonomic sample that included a wide representation of auditory specialists, the bats, did not uncover any evidence for a role in echolocation. We generated ‘conservation indices’ based on selection estimates at nucleotide sites and found that known disease mutations fall within sites of high evolutionary conservation. We suggest that methods such as this, derived from estimates of evolutionary conservation using phylogenetically divergent taxa, will help to differentiate between deleterious and benign mutations.

## Introduction

Sensory perception, including audition, plays one of the most important roles in the survival of an individual and is responsible for many key behaviours, for example, foraging, predator avoidance, mate recognition, and communication. These behaviours drive evolution and, therefore, it follows that genes involved in sensory perception should show signs of molecular adaptation. Given that mammals occupy many diverse environmental niches and rely on audition at different levels (MacDonald 2009), studying the molecular basis of this trait may help to elucidate loci that underpin different auditory capabilities. Through comparisons of phylogenetically diverse taxa with similar auditory capabilities, we may be able to uncover which ‘hearing’ genes show signatures of selection. Indeed, spectacular evidence of adaptive molecular convergence was recently reported in ‘hearing’ genes in echolocating whales and certain echolocating bats (Liu et al. 2010a, 2010b; Li et al. 2010, Davies et al. 2011), which has enabled a better understanding of the link between genotype and phenotype and, highlighted the utility of cross-species comparisons.

Phylogenomic comparisons across evolutionary divergent taxa can also uncover protein domains and residues, necessary to confer function (Kumar et al. 2011). Genic regions encoding such proteins will often be subject to strong purifying selection, and thus can be highly conserved over evolutionary timescales (Kumar et al. 2011; Dudley et al. 2012). Using this phylomedicine approach (Kumar et al. 2011), it is expected that disease-associated mutations (DAMs), particularly those implicated in Mendelian diseases, will be found within highly conserved genomic sites across phylogenetically and ecologically divergent taxa (Kumar et al. 2011; Dudley et al. 2012). Indeed, this rationale has been used to differentiate between disease and benign single-nucleotide variants (SNVs) in Freeman–Sheldon syndrome and Miller syndrome human cohorts (Cooper and Shendure 2011; Dudley et al. 2012). Identifying the so-called ‘*long-term evolutionary prior*’ of sites can help to predict whether particular mutations are likely to cause disease (Kumar et al. 2012). Therefore, to better predict disease association, it is important to locate these sites through cross-species comparisons and this can be done for any gene or genomic region.

**Figure 1:**
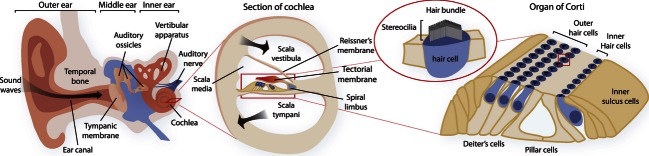
Illustration of the mammalian ear, detailing the overall structure of the complete ear, a cross-sectional view of the cochlea, the hearing component of the inner ear and the organ of Corti within, the location of sound transduction.

Hearing in mammals involves the conduction of a sound wave to the fluid-filled inner ear and transduction of this sound energy into neural impulses that are interpreted by the brain (Fig. 1). The part of the inner ear concerned with hearing is the cochlea: a tapering spiral-shaped organ encased in bone and containing three parallel tubes, with one of these, the scala media, wedged between the others (Brown et al. 2008). A sound wave generated in the scala media is picked up by the organ of Corti, running longitudinally along its base. Microvilli-like structures called stereocilia protrude from the apices of sensory ‘hair cells’, embedded in the organ of Corti (Fettiplace and Hackney 2006). The outer hair cells (OHCs), oscillate in response to the sound wave and deflect off a gelatinous structure called the tectorial membrane (TM) (Steel 2000). This triggers an action potential in these cells, causing the stereocilia of these cells to vibrate and thus amplifying the sound wave, a process known as cochlear amplification. In response to this, the inner hair cells (IHCs) oscillate and deflect off the TM, triggering a nerve impulse from these cells directed to the brain (Fig. 1). This system is considered one of the most intricate forms of sensation mechanisms in mammals (Dror and Avraham 2010).

Any hearing loss can have a profoundly negative effect on the quality of life of an individual. Hearing loss is the most common sensory deficit in humans with 1 in 1000 newborns being affected and over 50% of individuals over 80 years of age suffering from hearing impairment (Raviv et al. 2010). At least half of these cases are attributable to underlying genetic causes (Yan and Liu 2010). To date, 144 loci and 63 genes are known to be involved in human nonsyndromic hearing loss (NSHL), where deafness occurs without other symptoms (http://hereditaryhearingloss.org; Accetturo et al. 2010; Yan and Liu 2010; Lenz and Avraham 2011). This includes recessive, dominant, X- and Y-linked and mitochondrial mutations (Lenz and Avraham 2011). Although rapid advances have been made in documenting genes involved in deafness, there is still much to know about the molecular basis of normal auditory function (Brown et al. 2008) and little is known about the contribution of individual SNVs to disease. Given the potential and promise of therapeutic interventions in deafness (i.e. gene, stem cell and mechanical therapies), which optimally work when exactly matched to the underlying cause (Dror and Avraham 2010), it is imperative to better diagnose the genetic basis of hearing loss. To do this, it is essential to differentiate between disease and benign SNVs and elucidating the ‘*long-term evolutionary prior*’ of a site can enable a better prediction.

Here, we use cross-species comparisons of 11 human ‘hearing’ genes across evolutionary and ecologically divergent mammals, and use evolutionary methodologies to uncover regions of these genes that show: (i) parallel signatures of adaptation across mammals with similar hearing capabilities; and, (ii) regions of phylogenetic conservation within which we predict DAMs should occur. Our taxonomic representation is dominated by the mammalian acoustic specialists, the bats, which uniquely utilize sound to orient in complete darkness (i.e. echolocation; Teeling et al. 2012) and therefore, possess highly specialized auditory systems. We ascertain if our focal ‘hearing’ genes are also potential ‘echolocation genes’ and therefore, can illuminate the long-standing evolutionary question of whether echolocation was gained or lost in nonecholocating Old-World fruit bats (Teeling et al. 2009, 2012). Using a phylomedicine approach and deep phylogenetic sampling, we identify regions of these genes under strong purifying selection. We estimate the ‘*long-term evolutionary prior*’ of these sites using two different evolutionary methods and map human disease SNVs onto these sites to ascertain if these conserved sites are potential disease hotspots as we predicted.

## Methods

### Genes and Taxonomic Coverage

Eleven ‘hearing’ genes (*Myo15*, *Ush1 g*, *Strc*, *Tecta*, *Tectb*, *Otog*, *Col11a2*, *Gjb2*, *Cldn14*, *Kcnq4* and *Pou3f4*) were selected for comparative analysis of coding sequence evolution in eutherian mammals. These ‘hearing’ genes were chosen due to their involvement in the central processes of hearing based on at least one of the following criteria: (i) the presence of NSHL-associated mutations in the human orthologue; (ii) expression in the inner ear; and, (iii) interaction with known hearing genes and loss of hearing in knockout or sporadic mutant mice (criteria summarized in Tables S1 and S2). Selected gene sequences were downloaded from ensembl version 56 database (Hubbard et al. 2009) for 33 phylogenetically diverse mammals representing the major eutherian superordinal clades, with additional marsupial and tetrapod outgroups (Table S3). *Pou3f4* and *Tecta* (specifically the regions comprising exons 7, 9 and 18–19 of *TECTA* in human) were selected for further amplification and sequencing in additional eutherian taxa, focusing on the ‘hearing and auditory’ specialists, the bats (Chiroptera; Table S3). These genes were selected on the basis of suitability for Polymerase Chain Reaction (PCR) amplification given gene length, exon length, intron length, abundance of known human DAMS and potential role in echolocation.

### Downloaded Data Sets

Eutherian coding sequence data, homologous to the 11 human ‘hearing’ genes and of sufficient quality and coverage (i.e. did not contain major gaps or obvious sequence errors and could be readily aligned) were downloaded from ensembl version 56 (Hubbard et al. 2009) using blast release 2.2.22 (Altschul et al. 1997). clustal w2 release 2.0 (Larkin et al. 2007) was used to align the homologous sequence data, which was performed using mega 4.0 (Kumar et al. 2008). Alignment-ambiguous regions (highly variable regions of sequence which could not be satisfactorily aligned), were removed from the data sets in *Myo15*, *Otog* and *Cldn14*, while keeping the alignment in frame. For the 10 ‘downloaded only’ gene fragments (*Myo15*, *Ush1 g*, *Strc*, *Tecta*, *Tectb*, *Otog*, *Col11a2*, *Gjb2*, *Cldn14*, *Kcnq4*), data sets were constructed for each gene for up to 22 eutherian mammals (Eutherian alignments; Table S4). For *Myo15*, three separate data sets were assembled due to the length and discrete functional components of the motor protein: *Myo15* I, *Myo15* II and *Myo15* III, corresponding to the N-terminal domain, motor domain and the remaining C-terminal regions of the *MYO15* protein respectively.

### Amplified Data Set

Seventeen cross-species PCR primers (Table S5) were designed using parameters detailed in Bekaert and Teeling (2008) and primer3 (Rozen and Skaletsky 2000) to amplify regions of *Tecta* and *Pou3f4* in 38 mammals (Table S3). The taxa chosen for amplification and sequencing of homologous regions were primarily bats (20 echolocating bats from both subordinal clades, five nonecholocating pteropodid bats) and representatives of all other mammalian superordinal groups. Each PCR reaction was performed in a total volume of 25 μL containing 1× buffer (Life Technologies™, Carlsbad, CA, USA), 50 μmol/L deoxynucleotides (Life Technologies™), 1.5 mmol/L MgCl_2_ (Life Technologies™), 0.4 μmol/L of each primer (Life Technologies™), 1 U Platinum Taq (Life Technologies™) and 25 ng genomic DNA. Touchdown thermocycle reactions consisted of denaturation at 95°C for 5 min, 10 cycles of denaturing at 95°C for 30 s, annealing at 60°C for 30 s minus 1°C per cycle, extension at 72°C for 60 s, followed by 35 cycles with denaturation at 95°C for 30 s, annealing at 55°C for 30 s, extension at 72°C for 60 s and extension at 72°C for 10 min. The PCR products were separated and visualised by gel electrophoresis in a 1% agarose gel with SYBR® Safe DNA gel stain (Life Technologies™). Where reactions were unsuccessful or suboptimal, these were repeated with higher or lower annealing temperature or a different primer combination, if available. Where multiple bands were present, single bands of appropriate size were isolated using a pipette under UV light and these products were reamplified. Samples were then purified using either the ExoSap procedure or a Millipore filter plate (Millipore Corporation, Billerica, MA, USA). The ExoSap reaction was carried out using 0.05 μL Exonuclease I (at 10 U/μL) and 0.50 Shrimp alkaline phosphatase (at 1 U/μL) added to 4.45 μL ddH_2_O. These reagents were then added to each PCR product aliquot and incubated for 20 min at 37°C, then 5 min at 95°C and then resuspended in ddH_2_O. Sanger sequencing of the products was carried out by Macrogen (Seoul, Korea). Sequences were curated using Sequencher 4.8 (Gene Codes Corp., Ann Arbor, MI, USA).

Four aligned data sets (Chiroptera alignments; Table S4) were constructed representing the newly generated coding sequence for *Tecta* exon 7; *Tecta* exon 9; *Tecta* exons 18–19; *Pou3F4*. These data sets were used to ascertain the levels of selection on hearing genes specifically within the bat lineages (see below). These four data sets were also concatenated with: (i) the downloaded eutherian sequence data for relevant gene fragments (eutherian alignment; Table S4); and, (ii) with additional tetrapod homologous gene sequence data (tetrapod alignment; Table S4) and analysed to investigate signs of purifying/adaptive selection acting among sites and on the ancestral mammalian lineage (see below).

### Gene trees

For each of the eutherian mammal alignment data sets, phylogenetic trees were constructed using raxml version 7.0.4 (Stamatakis 2006; Stamatakis et al. 2008). raxml uses a likelihood-based tree-searching algorithm to find an optimal phylogeny using GTR-based models of site substitution – GTR + Γ models were used for these analyses. Bootstrapping (*n* = 100) was used to test the robustness of these phylogenies.

### Selection tests

To test for signatures of positive selection acting across eutherian mammals for each of the 11 hearing genes, we compared the likelihood scores of several selection models implemented in codeml in the paml package release 4.2 (Yang 2007) using likelihood ratio tests (LRTs). To examine selection across sites, alternative site models in which positive selection (*ω* > 1) was allowed to occur were compared to null models using a chi-square distribution. Two LRTs: model 7 vs model 8 (M7 vs M8) and model 8 vs model 8a (M8 vs M8a) (Swanson et al. 2003; Yang et al. 2005) were applied to: (i) the eutherian alignment for each data set; and, (ii) the Chiroptera alignment for *Tecta* exon 7, *Tecta* exon 9 and *Tecta* exons 18–19. These tests perform well in identifying positive selection in comparative alignments (Swanson et al. 2003; Yang et al. 2005). Model M7 assumes that *ω* follows a continuous beta distribution and serves as the null model in the M7 vs M8 LRT. M8 includes an extra site class, which allows *ω* ≥ 1 and two additional free parameters: *p*_*s*_, the proportion of codons with *ω* > 1, and *ω*_*s*_, the value of *ω* in these sites (Swanson et al. 2003). In the M8 vs M8a test, M8a is the null model and is identical to M8 except that *ω* cannot exceed one. One degree of freedom is used with the M8 vs M8a LRT. The M8 vs M8a test is robust to some of the potential difficulties associated with M7 vs M8 test, such as *ω* not conforming well to a beta distribution. Depending on the characteristics of the data, either test may be more powerful (Swanson et al. 2003). The Bayesian empirical Bayes (BEB) values (Yang et al. 2005) estimated from Model 8 were used to identify sites under significant positive selection.

Branch-site models were used to identify positive selection acting within bats versus other mammals using the *Tecta* exon 7, *Tecta* exon 9 and *Tecta* exons 18–19 data sets for the bat alignments. To ascertain selection acting on mammals, but absent in other tetrapods, the branch-site models were also run on the tetrapod alignment. The revised branch-site model A was used, which attempts to detect positive selection acting on a few sites on particular specified lineages – ‘foreground branches’ (Yang et al. 2005; Zhang et al. 2005). Ancestral branches of the clades of interest: (i) all bats; (ii) all mammals; and (iii) the bat subordinal divisions, Yinpterochiroptera and Yangochiroptera (Teeling et al. 2005), were categorized as ‘foreground branches’ in separate, independent tests. Four classes of sites are assumed in the model, and codons are categorized into these site classes based on foreground and background estimates of *ω*. Four free parameters in the *ω* distribution are estimated in the data. The alternative hypothesis that positive selection occurs on the foreground branches (*ω*_2_ > 1) is compared with the null hypothesis, where *ω*_2_ = 1 is fixed, using an LRT (Anisimova and Yang 2007). The two-ratio model test is also used for these comparisons. This test assumes that the foreground branch *ω* differs from the background *ω* by investigating positive selection operating on specific lineages (Yang 1998).

In total, 50 null hypothesis tests to identify positive selection, as described above, were carried out, representing 38 site-model tests (19 M7 vs M8 and 19 M8 vs M8a), six branch-site tests and six two-ratio tests. To account for multiple testing of related hypotheses on the same data set these null hypothesis tests have been grouped into families of related tests. The false-discovery rate procedure of Benjamini and Hochberg (1995) was then applied to all the members of a given hypothesis family. A total of 13 hypothesis families were defined. One family was defined for each of *Tectb*, *Otog*, *Col11a2*, *Strc*, *Ush1 g*, *Pou3f4*, *Kcnq4*, *Gjb2* and *Cldn14* (families A–I respectively). One family (J) was defined to represent *Myo15* (comprising the longest region: *Myo15* III). One family (K) representing the *Tecta* mammal analyses (14 hypothesis tests) whereas another (L) representing the *Tecta* bat analyses (12 hypothesis tests). A final family (M) was defined comprising the three regions of *Myo15*, to compare these regions with one another. The corrected *P*-values are displayed in parentheses below the uncorrected *P*-values in the results tables in the form *P*_*N*_ = *α*, where *N* is the hypothesis family and *α* is the respective false-discovery rate corrected *P*-value.

### Predicting DAMs

We predicted DAMs based on the idea of the ‘*evolutionary prior*’ (Kumar et al. 2011; Dudley et al. 2012). The eutherian mammal alignments of each data set were utilized to determine whether the evolutionary divergent homologous sequences could be used to accurately predict important diagnostic sites for human hearing loss, using a phylomedicine approach as described in Burk-Herrick et al. (2006).

Briefly, the calculated BEB values (Yang et al. 2005) were used to assign mean weighted *ω* values to all amino acid sites in the eutherian mammal alignments of the each data set. This was based on the category of selection that each site was under as estimated by BEB under model 8. The BEB values were used in all cases, even when the M7 vs M8 LRT was not significant; however, we only regard this as evidence of positive selection in the cases when the M7 vs M8 LRT was significant. These weighted mean *ω* values were used to produce an overall measure of conservation due to purifying selection on each amino acid site. A conservation index for each site undergoing purifying selection (*ω* < 1) was calculated as 1 − *ω* yielding a continuous spectrum of sites undergoing weak-to-strong purifying selection (0 < conservation index <1 respectively). The conservation index for sites undergoing neutral or positive selection (*ω* ≥ 1) was set at zero (Burk-Herrick et al. 2006). To determine whether the conservation indices derived from each of these data sets could yield significant site-specific measurements of purifying selection, estimates of minimum *ω* values were calculated for each translated codon for each data set. Minimum *ω* value categories were generated by successively adding posterior probabilities (PP) starting from the lowest *ω* class of BEB values. Each site was then categorized by the minimum *ω* value class at which a PP > 0.95 was attained. Data sets which included codon sites with significant evidence of negative (purifying) selection occurring (PP > 0.95 achieved in a category with a low omega value) were regarded as having the power to resolve purifying selection and were therefore deemed suitable to generate meaningful confidence indices of selection. Values of *ω* < 0.25 were taken as being low omega values and thus indicative of purifying selection. For example, if a codon has a significant value (PP > 0.95) in the first site class and this site class has a mean *ω* value of 0.1, this is evidence of strong purifying selection.

The location of known DAMs in each of the sequence alignments were identified using the UniProt Protein Knowledgebase database (Magrane and Consortium 2011). These locations were compared with the estimated conservation index values for each location based as described above. We used the online bioinformatic tool Polyphen 2 with default settings (Adzhubei et al. 2010) to additionally categorize the known NSHL DAMS. Polyphen 2 uses alignments of proteins from the UniProt database and protein structural characteristic to predict whether an amino acid substitution is deleterious or not (Adzhubei et al. 2010).

## Results

### Alignments

A total of 81 fragments were amplified and sequenced (GenBank accession Numbers: JF811703–JF811726; JF827767–JF827823; JX559619–JX559620) and concatenated with the downloaded data sets (Table S3). All of the gene sequences appeared functional on the basis that no nonsense mutations, insertions or deletions were detected. *Pou3f4*, *Tecta* exon 7, *Tecta* exon 9 and *Tecta* exons 18–19 were represented by 24, 20, 24 and 13 different bat species sequences respectively (Table S3).

### Gene trees

Gene trees with marsupial outgroups were produced for all eutherian mammal alignments. The consensus species tree (Teeling et al. 2005; Meredith et al. 2011) was predominantly recovered with high bootstrap support and little deviation. We found no evidence of sequence convergence in any of the gene trees, as unrelated lineages of echolocating bats and cetaceans were not seen to cluster together in a single clade. Nevertheless, all bats, including nonecholocating taxa, were monophyletic in all analyses with the exception of *Tecta* exon 7, in which the vespertilionid species (*Myotis lucifugus* and *Kerivoula pellucida*) grouped with the cetaceans with low support (bootstrap value = 39).

### Adaptive evolution in the mammals

The false-discovery rate corrected *P*-values for each of the 50 null hypothesis tests of adaptive evolution are indicated in the results tables (Tables 1–5), with respect to the hypothesis families in which they are included (see Methods). Significant positive selection was identified in placental mammals in *Myo15* (region III) and *Tecta* exon 9 alignments from the M8 vs M8a LRT and from both M7 vs M8 and M8 vs M8a LRTs in the case of *Otog* (Table 1). One site under significant positive selection was identified for the *Tecta* alignment at codon 830 (with *ω* of 1.476 ± 0.141 and PP of 0.969), corresponding to exon 9 of this gene, and the same site was recovered as significant (*ω* = 1.497 ± 0.197 and PP of 0.965) in the *Tecta* exon 9 alignment. The LRTs based on the site model parameters did not recover any evidence of positive selection in the remaining eutherian mammal alignments, including *Pou3f4*, which is taxonomically well represented. Comparison of the three regions of *Myo15* (*Myo15* I, *Myo15* II and *Myo15* III) did not reveal any significant evidence of positive selection on these regions following correction for multiple hypothesis testing (Table 5).

**Table 1:** Results for CODEML site model test of positive selection in eutherian (placental) mammals (*α* = 0.05); *p*_*s*_ signifies the proportion of sites with *ω* > 1; *ω*_*s*_ signifies the mean of these sites. False-discovery rate corrected *P*-values are included in parentheses. *P*_A__–__K_ refer to hypothesis families A–K respectively. Families A–J comprise two hypothesis tests each, whereas family K comprises 14 tests. Significance levels: ^*^P < 0.05, ^*^^*^p < 0.01

Region	d*N*/d*S*	Selection parameters	M7 vs M8	M8 vs M8a	Significant sites (model 8 Bayesian empirical Bayes)
*Tectb*	0.064	*p*_*s*_ = 0.002, *ω*_*s*_ = 1.000	*P* = 0.991 (*P*_A_ = 0.991)	*P* = 0.895 (*P*_A_ = 0.991)	–
*Otog*	0.170	*p*_*s*_ = 0.019, *ω*_*s*_ = 1.790	*P* = 0.034^*^ (*P*_B_ = 0.034^*^)	*P* = 0.009^*^^*^ (*P*_B_ = 0.018^*^)	–
*Col11a2*	0.103	*p*_*s*_ = 0.051, *ω*_*s*_ = 1.022	*P* = 0.482 (*P*_C_ = 0.482)	*P* = 0.227 (*P*_C_=0.454)	241 (*ω* = 1.486 ± 0.104, PP = 0.982)
*Strc*	0.202	*p*_*s*_ = 0.053, *ω*_*s*_ = 1.240	*P* = 0.204 (*P*_D_ = 0.204)	*P* = 0.075 (*P*_D_ = 0.15)	–
*Ush1g*	0.027	*p*_*s*_ = 1.0e−5, *ω*_*s*_ = 1.000	*P* = 0.999 (*P*_E_ = 0.999)	*P* = 0.371 (*P*_E_ = 0.742)	–
*Pou3f4*	0.051	*p*_*s*_ = 0.006, *ω*_*s*_ = 1.000	*P* = 0.732 (*P*_F_ = 0.732)	*P* = 0.413 (*P*_F_ = 0.732)	–
*Kcnq4*	0.031	*p*_*s*_ = 0.014, *ω*_*s*_ = 1.000	*P* = 0.058 (*P*_G_ = 0.116)	*P* = 0.331 (*P*_G_ = 0.331)	–
*Gjb2*	0.033	*p*_*s*_ = 0.008, *ω*_*s*_ = 1.000	*P* = 0.548 (*P*_H_ = 0.548)	*P* = 0.342 (*P*_H_ = 0.548)	–
*Cldn14*	0.044	*p*_*s*_ = 0.005, *ω*_*s*_ = 1.000	*P* = 0.455 (*P*_I_ = 0.584)	*P* = 0.584 (*P*_I_ = 0.584)	–
*Myo15* III	0.143	*p*_*s*_ = 0.066, *ω*_*s*_ = 1.000	*P* = 0.071 (*P*_J_ = 0.071)	*P* = 0.021^*^ (*P*_J_ = 0.042^*^)	–
*Tecta*	0.043	*p*_*s*_ = 0.010, *ω*_*s*_ = 1.000	*P* = 0.094 (*P*_K_ = 0.329)	*P* = 0.561 (*P*_K_ = 0.873)	830 (*ω* = 1.476 ± 0.141, PP = 0.969)
*Tecta* exon 7	0.053	*p*_*s*_ = 0.006, *ωs* = 1.189	*P* = 0.409 (*P*_K_ = 0.716)	*P* = 0.389 (*P*_K_ = 0.716)	–
*Tecta* exon 9	0.047	*p*_*s*_ =0.020, *ωs* = 1.405	*P* = 0.010^*^^*^ (*P*_K_ = 0.07)	*P* = 0.002^*^^*^ (*P*_K_ = 0.028^*^)	830 (*ω* = 1.497 ± 0.197, PP = 0.965)
*Tecta* exons 18–19	0.026	*p*_*s*_ = 1.0e − 5, *ωs* = 2.659	*P* = 0.999 (*P*_K_ = 1.000)	*P* = 0.974 (*P*_K_ = 1.000)	–

**Table 2:** Results for CODEML revised model A branch-site test and two-ratio test of positive selection in *Tecta* regions acting on the mammals versus other tetrapods (*α* = 0.05). False-discovery-rate-corrected *P*-values are included in parentheses. *P*_K_ refers to hypothesis family K, which comprises 14 tests

*Tecta* region	Revised model A branch-site test		Two-ratio test

	Significant codon sites
*Tecta* exon 7	*P* = 0.087 (*P*_K_ = 0.329)	68 (PP = 0.974); 162 (PP = 0.996)	*P* = 0.943 (*P*_K_ = 1.000)
*Tecta* exon 9	*P* = 0.144 (*P*_K_ = 0.373)	2 (PP = 0.981), 20 (PP = 0.980)	*P* = 0.160 (*P*_K_ = 0.373)
*Tecta* exons 18–19	*P* = 1.000 (*P*_K_ = 1.000)	–	*P* = 0.780 (*P*_K_ = 1.000)

**Table 3:** Results for CODEML site model test of positive selection in *Tecta* regions in the bat clade (*α* = 0.05); *p*_*s*_ signifies the proportion of sites with *ω* > 1; *ω*_s_ signifies the mean of these sites. False-discovery-rate-corrected *P*-values are included in parentheses. *P*_L_ refers to hypothesis family L, which comprises 12 tests. ^*^Significant result at P < 0.05

*Tecta* region	d*N*/d*S*	Selection parameters	*P*-value		Significant sites (model 8 Bayesian empirical Bayes)

M7 vs M8	M8 vs M8a
*Tecta* exon 7	0.066	*p*_*s*_ = 0.0176, *ω*_*s*_ = 2.1209	*P* = 0.053 (*P*_L_ = 0.318)	*P* = 0.015^*^ (*P*_L_ = 0.18)	407 (*ω* = 2.12 ± 0.828, PP = 0.958)
*Tecta* exon 9	0.059	*p*_*s*_ = 0.0494, *ω*_*s*_ = 1.1680	*P* = 0.323 (*P*_L_ = 0.969)	*P* = 0.133 (*P*_L_ = 0.532)	–
*Tecta* exons 18–19	0.005	*p*_*s*_ = 1.0e − 5, *ω*_*s*_ = 1.0000	*P* = 0.999 (*P*_L_ = 1.000)	*P* = 0.753 (*P*_L_ = 1.000)	–

**Table 4:** Results for CODEML revised model A branch-site test and two-ratio test of positive selection in *Tecta* regions acting on the Eutheria versus Chiroptera (*α* = 0.05). False-discovery-rate-corrected *P*-values are included in parentheses. *P*_L_ refer to hypothesis family L, which comprises 12 tests

*Tecta* region	Revised model A branch-site test	Two-ratio test
	
	*P*-value	*P*-value
Exon 7	*P* = 1.000 (*P*_L_ = 1.000)	*P* = 0.998 (*P*_L_ = 1.000)
Exon 9	*P* = 1.000 (*P*_L_ = 1.000)	*P* = 0.508 (*P*_L_ = 1.000)
Exons 18–19	*P* = 1.000 (*P*_T_ = 1.000)	*P* = 0.780 (*P*_T_ = 1.000)

**Table 5:** Results for CODEML site model test of positive selection in eutherian (placental) mammals in the three regions of *Myo15* (*α* = 0.05); *p*_*s*_ signifies the proportion of sites with *ω* > 1; *ω*_s_ signifies the mean of these sites. False-discovery-rate-corrected *P*-values are included in parentheses. *P*_M_ refer to hypothesis family M, which comprises 6 tests. ^*^Significant result at P < 0.05

*Myol 5* region	d*N*/d*S*	Selection parameters	*P*-value		Significant sites (model 8 Bayesian empirical Bayes)

M7 vs M8	M8 vs M8a
*Myo15* I (N-terminal)	0.174	*p*_*s*_ = 0.025, *ω*_*s*_ = 1.776	*P* = 0.038^*^ (*P*_M_ = 0.152)	*P* = 0.011^*^ (*P*_M_ = 0.066)	–
*Myo15* II (Motor)	0.050	*p*_*s*_ = 0.008, *ω*_*s*_ = 1.000	*P* = 0.828 (*P*_M_ = 1.000)	*P* = 0.630 (*P*_M_ = 1.000)	–
*Myo15* III (C-terminal)	0.143	*p*_*s*_ = 0.066, *ω*_*s*_ = 1.000	*P* = 0.071 (*P*_M_ = 0.213)	*P* = 0.021^*^ (*P*_M_ = 0.105)	–

None of the branch-site (revised model A) LRTs showed any evidence of positive selection acting on mammals in any of the three data sets (*Tecta* exon 7, *Tecta* exon 9, *Tecta* exon 18–19; Table 2). It is also possible that the background branches (the nonmammal tetrapods) may have themselves undergone some periods of positive selection and therefore, may not be completely conserved. The two-ratio (branch) test did not find significant evidence of positive selection in any of the four data sets. A significant *P*-value in this test would indicate positive selection in mammals that is absent in the other tetrapods.

### Adaptive evolution in the bats

The results of one of the site-model tests (M8 vs M8a) of the bat alignments provided evidence of positive selection acting on the *Tecta* gene in the bats – specifically in exon 7 (Table 3), although this was not evident from the other LRT (M7 vs M8). One codon was identified as undergoing significant positive selection by BEB corresponding to codon 407 of the human orthologue (*ω* = 2.12 ± 0.828, PP = 0.958). Branch and branch-site tests reveal no signature of positive selection acting at the base of Chiroptera (Table 4). The branch-site test did not recover positive selection acting on the ancestral yangochiropteran nor yinpterochiropteran branches for *Tecta* exon 7 or *Tecta* exon 9. Nonsignificant *P*-values for the LRTs of *Tecta* exon 18–19 for the bat alignments indicate no evidence for positive selection in this clade and suggest that this region is conserved in bats, as in eutherian mammals as a whole.

### Predicting DAMs

**Figure 2:**
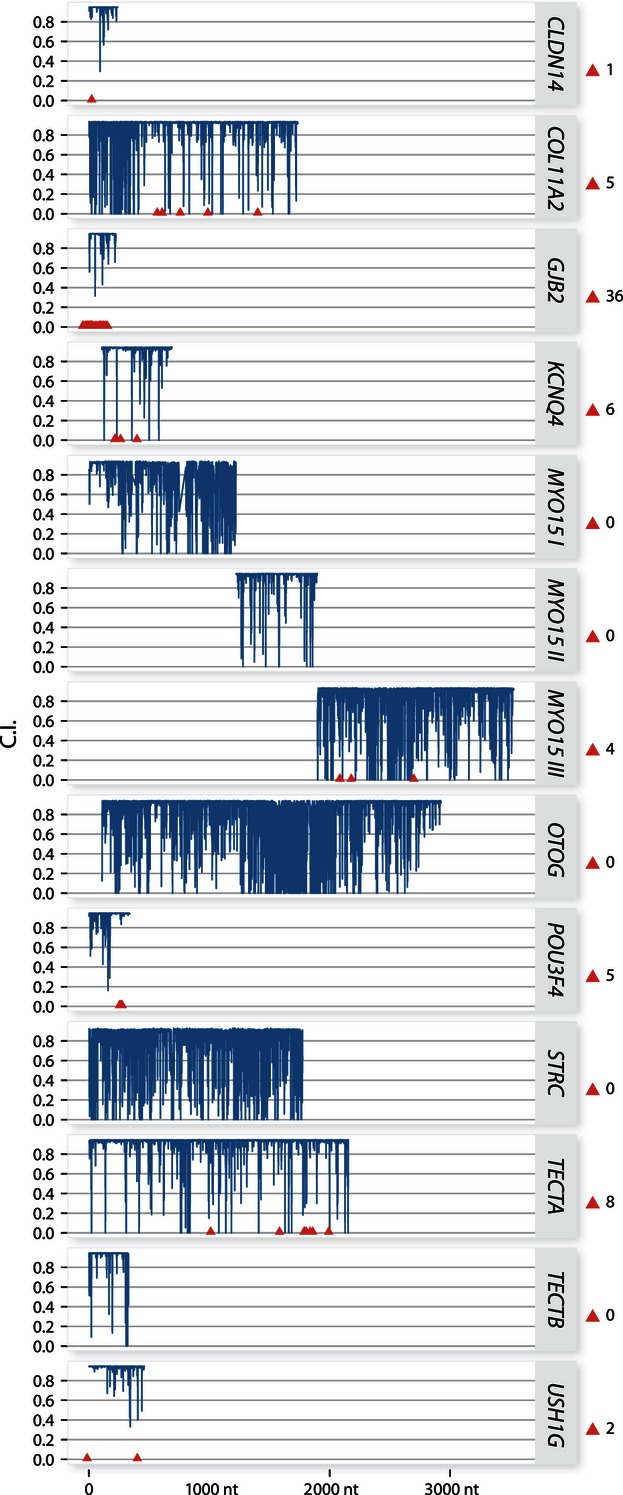
Conservation index values, derived from alignments of eutherian mammal taxa, for each gene in this study (*Myo15* is divided into three alignments: *Myo15* I, II and III). The location of known disease-associated mutations (DAMs) are indicated with red triangles.

**Figure 3:**
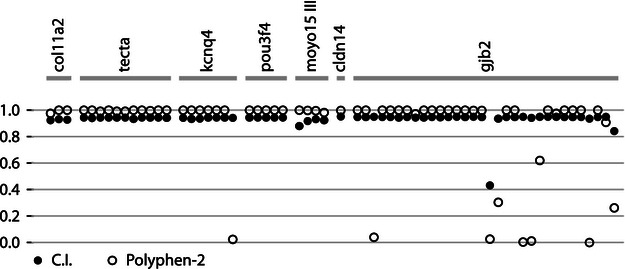
Conservation index values and Polyphen-2 scores for each known NSHL-associated missense SNV in this study (both sets of values are graded on a scale from 0 to 1). The genes within which the DAMs are located (*n* = 7) are indicated. NSHL, nonsyndromic hearing loss; SNV, single-nucleotide variant, DAM, disease-associated mutation.

The confidence indices and the *ω* value for every codon position for each gene fragment were estimated (Fig. 2), establishing the ‘*evolutionary prior’* of each position. As expected, BEB results revealed high rates of purifying selection associated with regions thought to correspond to known conserved domains. This was best illustrated by the homeobox region of *Pou3f4,* but was also evident in the core region of *Kcnq4*. Reflecting the signature of high purifying selection predominantly identified by the LRTs and BEB values, the conservation index indicates high rates of purifying selection across eutherian mammals (Fig. 2). High PP values for categories of selection with very low mean *ω* (<0.001) were present for each of the alignments. This provides strong evidence that the high conservation index values indicated are robust and not simply indicative of low sequence divergence or poor taxonomic representation. Sixty-one NSHL DAMs were identified from UniProt and mapped onto each gene fragment (Fig. 3). As predicted, all but three of the 61 DAM sites have a conservation index of ≥0.9, so are highly conserved. Polyphen 2 categorized these 61 DAMs as probably damaging in 52 cases, possibly damaging in two cases and benign in six cases (Fig. 3).

## Discussion

### Hearing genes are predominantly evolutionarily conserved in mammals

Our results identify putative positive selection in three (*Myo15*, *Otog* and *Tecta*) of the 11 genes examined, but cannot in all cases rule out the possibility that positive selection is acting on the remaining genes in mammal taxa. In some cases, such as the transcription factor gene *Pou3f4*, the clear absence of evidence of positive selection acting on placental mammals in the tests applied strongly suggests that these genes are not undergoing adaptive evolution in these taxa, but are predominantly evolutionary conserved and are undergoing purifying selection. In the three genes where signals of positive selection have been identified, positive selection is localized in particular regions. Mammals are heavily reliant on hearing for a variety of ecologically important roles (Macdonald 2009) and it is likely that evolution has acted primarily to conserve the function of hearing genes, many of which are functionally conserved in tetrapods. There are no known naturally ‘deaf’ mammals (Macdonald 2009; Horowitz 2012) suggesting the important role of hearing in mammalian survival; therefore, gene conservation is to be expected. The less conserved regions which do occur, including repeat regions in *Myo15* and *Cldn14*, are difficult to investigate comparatively because they cannot be readily aligned. Nonetheless, repeat regions often code functionally important components of a peptide and can be important sites of adaptive selection.

### Adaptive selection on mammalian hearing genes

#### Myo15

Detected molecular adaptation in the gene encoding the protein *Myo15* is interesting given its role in the development or maintenance of the stereocilial bundle (Belyantseva et al. 2005; Delprat et al. 2005). In particular, conformational changes to the protein product may affect its role in the transport of the protein whirlin to the stereociliary tip, which is important in the organization of stereociliary proteins and actin polymerization (Belyantseva et al. 2005). Nal et al. (2007) found that isoform 1 of Myosin-XV, which includes the N-terminal extension, is required for hearing in humans, based on their identification of 16 recessive NSHL mutations in human *MYO15A*. Further study is required to quantify the extent of positive selection occurring and its precise location within this gene.

#### Otog

The pattern of *ω* exhibited by *Otog* in the eutherian mammals (derived from the BEB) indicates that purifying selection predominates across the majority of the sequence, but clearly identifies a signature of positive selection, localized to the region corresponding to the mucin domain of *Otog*. This suggests strong conservation of function acting on most of the gene, but adaptive evolution acting on virtually the entire mucin domain-coding region, which may be linked to the putative role of Otogelin in organization of the TM or potentially a role in the vestibule (Simmler et al. 2000). However, as mucin domain-containing proteins are frequently identified as undergoing adaptive evolution in selection studies, these are sometimes regarded as false positives. Therefore, we must be cautious in drawing inferences based on this result.

#### Tecta

Myo15 The overall image which emerges from analysing the *Tecta* data (the *Tecta*, *Tecta* exon 7, *Tecta* exon 9 and *Tecta* exons 18–19 data sets) is of a gene that is primarily under a high degree of purifying selection, but with highly localized positive selection occurring on a small number of sites. This is consistent with a gene that includes numerous sites known to be associated with NSHL and whose protein product is highly conserved, containing numerous conserved domains. Conserved domains include the functionally important ZP domain – within which most of the known NSHL mutations are found. The absence of evidence for positive selection in the analyses conducted on the *Tecta* exons 18–19 data set reflects the location of this region – corresponding to a portion of the ZP domain. Even within the eutherian alignment of *Tecta* exon 9, the evidence for positive selection, although strong enough to produce a significant result in the M8 vs M8a LRT test – is essentially limited to three sites. These sites were found in a region corresponding to a von Willebrand factor type D domain, whereas the majority of other sites fell into the lowest category of omega (ω = 0) with significant PP (i.e. highly evolutionarily conserved). Longer gene fragments would be useful to elucidate more fully the selection pressures acting across mammals at this locus.

### Positive selection acting within bats

A number of recent studies have identified several putative ‘echolocation’ genes (*Prestin*, *Tmc1*, *Pjvk*, *Otof*, *Cdh23*, *Pcdh15*, *Kcnq4*) that each show varied levels of sequence convergence between the two main clades of laryngeal echolocating bats (Li et al. 2008; Davies et al. 2011; Liu et al. 2011, 2012; Shen et al. 2012). Remarkably, with the exception of *Kcnq4*, these genes were also reported to show evidence of sequence convergence between echolocating bats and echolocating toothed whales (Liu et al. 2010a; 2010b; Davies et al. 2011; Shen et al. 2012). In many cases, such convergence was so strong that it led to conflicts between the species phylogeny and gene trees based on the coding sequences, with erroneous clustering of distantly related echolocating taxa. The findings of these earlier studies also suggested that convergent substitutions were adaptive on the basis of associations with positive selection as well as with hearing sensitivity in echolocating taxa. Although the precise auditory roles of some of these genes remain unclear, many are involved with sensory hair cell development and/or function.

In contrast, phylogenetic analyses of our focal genes, including *Kcnq4*, did not show any major deviations from the consensus best supported mammalian topologies (Teeling et al. 2005; Meredith et al. 2011). Branch-site tests did not support any evidence of positive selection acting on the echolocating lineages within these genes. The only evidence for strong positive selection acting within the bats appears to occur at one site in *Tecta* exon 7. These results suggest that our focal genes are not prime ‘candidate’ echolocation genes and therefore cannot address the gain or loss of echolocation in the Old World Fruit bats. However, Liu et al. (2011, 2012) found evidence of parallel substitutions in specific regions of *Kcnq4* within echolocating bats and positive selection in the ancestral lineage leading to mammals. The smaller taxonomic representation in this study for this locus, particularly of echolocating lineages (one echolocating bat, one dolphin) must have been insufficient to recover evidence of this convergent adaptation. This suggests that in our ‘downloaded only’ data sets, a wider taxonomic representation of the hearing specialists may be needed to truly ascertain if they are ‘echolocation’ genes. However, this is not the case within our ‘amplified data sets’, given the taxonomic representation of bats and cetaceans. Therefore, we consider it unlikely that *Pou3f4* or the focal exonic region in *Tecta* play a significant role in ultrasound hearing. It would be interesting to further explore whether episodic events of selection have occurred in these data sets using emerging selection analyses (e.g. HyPhy; Murrell et al. 2012).

### Prediction of DAMs

The DAM sites predicted on the basis of conservation indices (‘*long-term evolutionary priors*’) constitute the first estimates of deafness-causing mutations using phylogenetically and ecologically diverse taxa. Such predictions will be invaluable in disease screening, enabling better diagnoses of the underlying genetic predisposition, driving the benefits of personal genome sequencing and personalized molecular medicine (Dudley et al. 2012). A major limiting factor in estimating ‘*evolutionary priors’* is taxonomic representation. This will soon to be overcome given the promise of ongoing large vertebrate sequencing projects such as Genome 10K (Genome 10K Community of Scientists 2009). The classification of most of the NSHL DAM sites as highly evolutionarily conserved, by the Burk-Herrick et al. (2006) method, illustrates its potential to recover true-positive (DAM) results and therefore predict where unidentified DAMs should fall. Polyphen 2 and other methods (such as SIFT (Kumar et al. 2009) and Mutation-Assessor (Reva et al. 2011; Kumar et al. 2012) utilize cross-species alignments of amino acid data to identify deep-level phylogenetic conservation (as well as characteristics of protein structure) and therefore predict which sites are likely to be deleterious. By utilizing all of the nucleotide data and basing the conservation indices on direct estimates of selection, the method of Burk-Herrick et al. (2006) adds a further dimension to these analyses. Each existing classification method has advantages and drawbacks and it has been shown that several different methods combined can achieve better results than independent analyses as demonstrated by the consensus method Condel (Kumar et al. 2012). We suggest that a method utilizing nucleotide data to predict the occurrence of DAMs, such as the conservation indices used, could augment the current arsenal of DAM-predicting techniques, and improve the utility of cross-species alignments for predicting deleterious mutations.

## Conclusion

We find putative evidence of adaptive evolution acting on eutherian mammals in the hearing genes *Myo15* and *Otog* and *Tecta*, and in *Tecta* acting on bats. We find little evidence of adaptive selection in bats suggesting that our focal genes are not ‘echolocation’ genes. By estimating ‘*evolutionary priors*’ of amino acid sites, we have predicted and confirmed the location of NSHL DAMs. We suggest that this methodology can be used to further develop accurate molecular diagnostics of auditory disease in man.
